# Autoantibodies to Apolipoprotein A-1 as Independent Predictors of Cardiovascular Mortality in Renal Transplant Recipients

**DOI:** 10.3390/jcm8070948

**Published:** 2019-06-29

**Authors:** Josephine L.C. Anderson, Sabrina Pagano, Julien Virzi, Robin P.F. Dullaart, Wijtske Annema, Folkert Kuipers, Stephan J.L. Bakker, Nicolas Vuilleumier, Uwe J.F. Tietge

**Affiliations:** 1 Department of Pediatrics, University Medical Center Groningen, University of Groningen, 1205 Groningen, The Netherlands; 2 Division of Laboratory Medicine, Department of Genetics and Laboratory Medicine, Geneva University Hospital, 1205 Geneva, Switzerland; 3 Department of Medical Specialties, Faculty of Medicine, Geneva University, 1205 Geneva, Switzerland; 4 Department of Endocrinology, University Medical Center Groningen, University of Groningen, 9713 GZ Groningen, The Netherlands; 5 Institute of Clinical Chemistry, University Hospital of Zurich and University of Zurich, 8006 Zurich, Switzerland; 6 Laboratory Medicine, University Medical Center Groningen, University of Groningen, 9713 GZ Groningen, The Netherlands; 7 Department of Nephrology, University Medical Center Groningen, University of Groningen, 9713 GZ Groningen, The Netherlands; 8 Division of Clinical Chemistry, Department of Laboratory Medicine H5, Karolinska Institutet, 14183 Stockholm, Sweden; 9 Clinical Chemistry, Karolinska University Laboratory, Karolinska University Hospital, SE-141 86 Stockholm, Sweden

**Keywords:** biomarker, anti-apolipoprotein A-1 antibodies, renal transplant recipient, HDL function, prognosis, cholesterol

## Abstract

Renal transplant recipients (RTRs) are known to have a high cardio-vascular disease (CVD) burden only partly explained by traditional CVD risk factors. The aim of this paper was therefore to determine: i) the prognostic value of autoantibodies against apoA-1 (anti-apoA-1 IgG) for incidence of CVD mortality, all-cause mortality and graft failure in RTR. Four hundred and sixty two (462) prospectively included RTRs were followed for 7.0 years. Baseline anti-apoA-1 IgG were determined and associations with incidence of CVD mortality (*n* = 48), all-cause mortality (*n* = 92) and graft failure (*n* = 39) were tested. Kaplan–Meier analyses demonstrated significant associations between tertiles of anti-apoA-1 IgG and CVD mortality (log rank test: *p* = 0.048). Adjusted Cox regression analysis showed a 54% increase in risk for CVD mortality for each anti-apoA-1 IgG levels standard deviation increase (hazard ratio [HR]: 1.54, 95% Confidence Interval [95%CI]: 1.14–2.05, *p* = 0.005), and a 33% increase for all-cause mortality (HR: 1.33; 95%CI: 1.06–1.67, *p* = 0.01), independent of CVD risk factors, renal function and HDL function. The association with all-cause mortality disappeared after excluding cases of CVD specific mortality. The sensitivity, specificity, positive predictive value, and negative predictive value of anti-apoA-1 positivity for CVD mortality were 18.0%, 89.3%, 17.0%, and 90.0%, respectively. HDL functionality was not associated with anti-apoA-1 IgG levels. This prospective study demonstrates that in RTR, anti-apoA-1 IgG are independent predictors of CVD mortality and are not associated with HDL functionality.

## 1. Introduction

Impaired kidney function is a major risk factor for cardiovascular diseases (CVD) through all stages of renal dysfunction, amounting to a substantial 40-fold increased risk of CVD mortality in end-stage renal disease (ESRD) patients [1,2]. Even after renal transplantation the CVD risk remains four to six times higher in age-adjusted analyses, with half of all deaths of renal transplant recipients (RTRs) being attributable to a CVD origin [3]. Traditional CVD risk factors, such as the ones aggregated in the Framingham risk score (FRS), are known to be of little use in CVD risk prediction in RTRs [4,5]. Thus, accurate CVD risk stratification in RTRs represents an unmet clinical need in this constantly increasing patient population.

Autoantibodies against apoA-1 (anti-apoA-1 IgG) represent a recently identified biomarker with a high potential to predict increased CVD risk. Increased levels of these antibodies are associated with a pro-atherogenic lipid profile, a systemic pro-inflammatory state [6,7,8], as well as high-density lipoprotein (HDL) dysfunction [9,10], and were shown to be associated with increased CVD risk and poorer prognosis in high-risk patients, as well as in the general population [7,11,12,13,14]. When administered to mouse models of atherosclerosis, anti-apoA-1 IgG enhanced atherogenesis, myocardial necrosis and premature death indicating that anti-apoA-1 IgG have the potential to serve as a causative biomarker for CVD [15,16,17]. A previous study showed that ESRD patients have a high prevalence of elevated anti-apoA-1 IgG levels, which was associated with dialysis vintage, and was a major determinant of cardiovascular outcomes in these patients [18]. The reasons underpinning such association in ESRD are still elusive, but may suggest that a prolonged exposure to the uremic milieu, characterized by increased oxidative stress and inflammation, could increase apoA-1 immunogenicity leading to an anti-apoA-1 IgG response. Accordingly, as RTR are exposed to a uremic milieu prior to transplantation, they could also constitute a particularly risk-prone group to such a humoral autoimmunity phenomenon, despite receiving immunosuppressive treatment.

The aim of our present study was to determine: i) the prognostic value of anti-apoA-1 IgG for incidence of CVD specific mortality, overall mortality and graft failure in RTR and ii) to delineate the relationship of anti-apoA-1 IgG with apoA-1 levels and HDL functionality.

## 2. Experimental Section

### 2.1. Study Design and Study Population

This study included all adult RTR who visited the University Medical Centre Groningen (UMCG) outpatient clinic between August 2001 and July 2003 with a functioning renal graft for at least 1 year. Of 847 eligible patients, 606 consented to participate in the overall study. Exclusion criteria consisted of congestive heart failure, malignant disease other than cured skin cancer, as well as endocrine abnormalities other than diabetes, or suspected acute infection upon inclusion, indicated by a CRP value above 15 mg/L. This way 477 patients were initially included in the present study; serum was available from 462 RTRs, in which subsequently anti-apoA-1 IgG levels were measured. All relevant patient characteristics were obtained from the “Groningen Renal Transplant Database”. Patients were followed for a period of 7 years, and no patients were lost during follow-up. More detailed definitions of the characteristics of the database patients’ baseline characteristics, as well as the routine laboratory methods used have been previously described [19,20]. The study was approved by the local Medical Ethics Committee (METc2001/039), and is in accordance with the Declaration of Helsinki and Principles of the Declaration of Istanbul as outlined in the ’Declaration of Istanbul on Organ Trafficking and Transplant Tourism’.

### 2.2. Outcome Measures

The main outcome measure in this study is the level of anti-apoA-1 IgG. The primary endpoints consisted in CVD mortality, all-cause mortality and graft failure. As previously reported [21,22]. graft failure was defined as return to dialysis or re-transplantation. Cause of death was obtained by linking the number on the death certificate to the primary cause of death, as coded by a physician from the Central Bureau of Statistics. CVD mortality was defined as deaths in which the principal cause of death was cardiovascular in nature, using ICD-9 codes 410–447. The secondary endpoint was a possible association between anti-apoA-1 IgG levels and apoA-1 levels, as well as a key HDL functionality, namely macrophage cholesterol efflux capacity (CEC).

### 2.3. Sensitivity Analyses

Sensitivity analyses were performed, in which the association of anti-apoA-1 IgG with non CVD-mortality was assessed, in order to assess the specificity of anti-apoA-1 with CVD.

### 2.4. Determination of Anti-apoA-1 IgG

Anti-apoA-1 IgG were measured using RTR serum aliquots stored at −80 °C, as previously described [6,7,8,11,12,13]. The experiments demonstrating the specificity of our assay against the native and unmodified form of apoA-1 are available in the Supplementary Materials.

### 2.5. Determination of HDL Function

To determine HDL-mediated CEC, a previously published method was used [21,22]. For further details see the Supplementary Materials.

### 2.6. Statistical Analysis

In order to eliminate bias due to gender specific differences in levels of anti-apoA-1 IgG renal transplant recipients were divided into gender-stratified tertiles based on levels of anti-apoA-1 IgG. This was done by first dividing the group into males and females, then computing tertiles based on the levels of anti-apoA-1 IgG, and subsequently merging the groups back together. Differences between baseline characteristics were tested. For continuous variables with a skewed distribution differences were tested by Kruskal–Wallis test. Differences for normally distributed continuous variables were tested by one-way analysis of variance followed by Bonferroni post-hoc test. Differences in categorical data were tested by chi-squared test.

Thereafter, multivariable linear regression analysis was performed to evaluate which variables predict levels of anti-apoA-1. Baseline characteristics with a *p*-value of ≤0.2 between tertiles of anti-apoA-1 IgG were first fitted into a univariate linear regression. Variables that had a significant association with anti-apoA-1 IgG in a univariate analysis were then entered into a multivariate linear regression. 

The association of anti-apoA-1 IgG levels with the primary endpoints was assessed by the log-rank test and by Cox proportional hazards regression. Kaplan-Meier curve analyses were performed across anti-apoA-1 IgG tertiles and according to anti-apoA-1 IgG seropositivity, based upon a predefined and validated anti-apoA-1 IgG cut-off value (an OD value >0.64 and a percentage of the positive control above 37%) [7,8,11,12,13,14,18]. Differences were assessed using a log-rank test. Cox regression analyses were used to calculate hazard ratios (HR) and reported with their 95% confidence intervals (95%CI). Univariate and multivariate Cox regression analyses were performed per standard deviation (SD, 0.316) increase of anti-apoA-1 IgG levels, and according to anti-apoA-1 IgG seropositivity. Multivariate analyses were performed using different models, taking into account traditional CV risk factors, renal function, HDL functionality, and all the variables that had significant association with anti-apoA-1 levels in linear regression. Schoenfeld residuals test was used to test the proportional hazard assumption for the outcomes of CVD mortality, all-cause mortality and graft failure for analysis per standard deviation increase (*p* = 0.18, *p* = 0.20 and *p* = 0.69 respectively) and for analysis with seropositivity (*p* = 0.38, *p* = 0.09 and *p* = 0.77 respectively), and was found not to be violated. Sensitivity (SN), specificity (SP), positive predictive and negative predictive values (PPV and NPV, respectively) for anti-apoA-1 IgG seropositivity were computed. *p*-values <0.05 were considered statistically significant. All statistical analyses were performed using the Statistical Package for the Social Sciences version 24 (IBM SPSS, IBM Corporation, Armonk, NY, USA) and GraphPad Prism version 6.0 (GraphPad Software, San Diego, CA, USA).

## 3. Results

### 3.1. Baseline Demographic Characteristics

In this longitudinal follow-up study the levels of anti-apoA-1 IgG were measured in 462 RTR. Of these patients, 92 (20%) died within the follow-up of 7 years, 48 of these from a confirmed CVD cause, as determined by ICD-9 codes 410–447 (10% of included patients, 52% of all recorded deaths). A total of 39 (8%) patients experienced graft failure. Overall, the prevalence of high levels of anti-apoA-1 IgG (anti-apoA-1 IgG seropositivity according to a previously defined cut-off value based on data from the general population) was 11.5 % (53/462). [7,11,12,13,18] In order to better explore the architecture of anti-apoA-1 IgG in RTR, patients were divided into gender-stratified tertiles of anti-apoA-1 IgG, with median values of 0.15 (range 0–0.24), 0.31 (range 0.24–0.49), and 0.64 (range 0.50–2.09) for the first, second and third tertile, respectively (Table 1). Analyses between tertiles showed a significant difference for the history of myocardial infarction (MI), which was most common in patients with the highest levels of anti-apoA-1 IgG (*p* = 0.047), as well as for diabetic nephropathy (*p* = 0.04) as the primary renal disease. A trend was also observed for tubulo-interstitial disease (*p* = 0.05), which is characterised by acute or chronic inflammation of the renal tubules and interstitium, and primary glomerular disease (*p* = 0.08), which covers a group of conditions in which there is primary injury in the glomerulus [23].

When participating RTR were stratified according to anti-apoA-1 IgG seropositivity, there was again a significant association with a higher prevalence of previous MI (*p* = 0.02), glomerular disease (*p* = 0.03) and tubulo-interstitial disease (*p* = 0.03) as the primary renal disease (Supplementary Table 1). Furthermore, anti-apoA-1 IgG seropositive patients tended to have received grafts from older donors (*p* = 0.05) and showed a higher urinary protein excretion (*p* = 0.04, Supplementary Table S1). Importantly, cholesterol efflux capacity as central HDL function metric did not differ between patients seropositive for anti-apoA-1 IgG and those seronegative for these antibodies (Supplementary Table S1). 

Subsequently, univariate and thereafter multivariate linear regression was performed to deduce which variables are independently associated with levels of anti-apoA-1 IgG (Table 2). A positive, independent association was seen between anti-apoA-1 IgG and a history of myocardial infarction (β = 0.103, *p* = 0.026) and primary glomerular disease (β = 0.116, *p* = 0.016). A negative association was seen with tubule-interstitial disease (β = −0.106, *p* = 0.028). No significant relationship between anti-apoA-1 IgG and the metric of HDL function, CEC, was discernible, nor with concentrations of HDL-C or with apoA-1. There was also no association with immunosuppressive drugs, either individually or combined.

### 3.2. Association with Incidence of CVD Mortality, All-Cause Mortality, and Graft Failure

**Figure 1:**
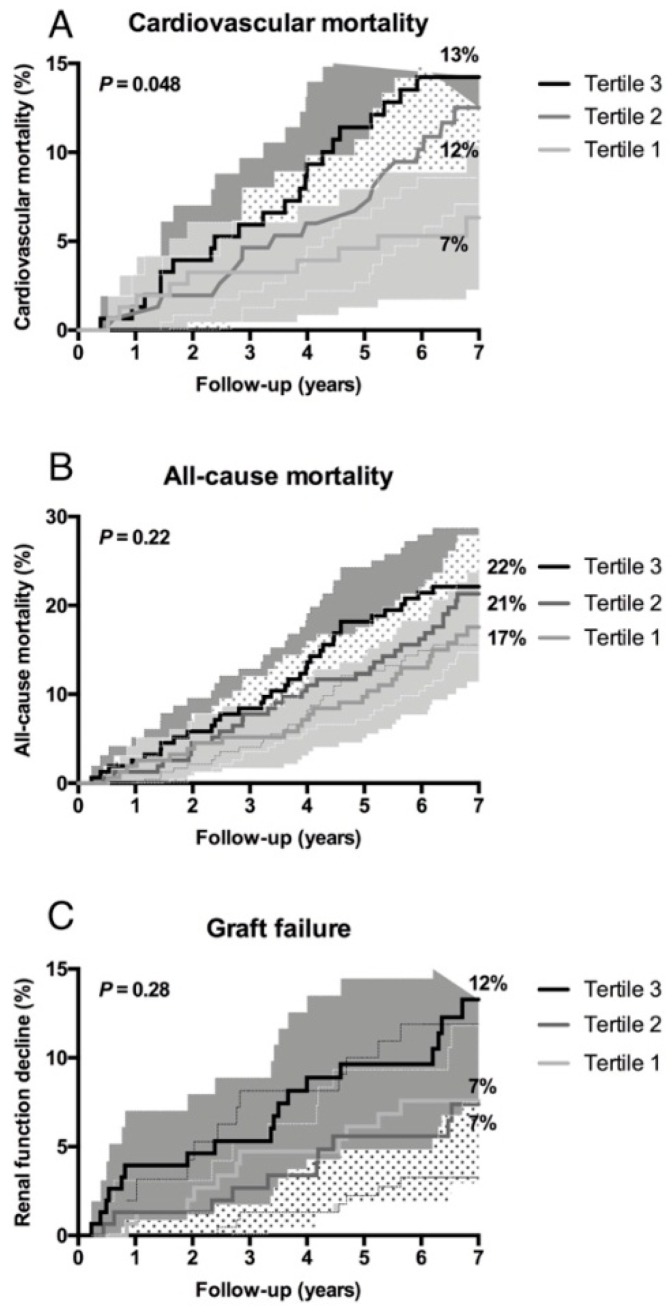
Higher levels of anti-apoA-1 IgG are associated with an increased incidence of cardiovascular mortality and all-cause mortality in renal transplant recipients. Kaplan-Meier curves depicting (**A**) cardiovascular mortality, (**B**) all-cause mortality, and (**C**) graft failure according to tertiles of anti-apoA-1 IgG. The corresponding P value was obtained from log-rank tests.

As shown in Figure 1, Kaplan Meier curves showed a significant association of tertiles of anti-apoA-1 IgG with CVD mortality (*p* = 0.048), but not with all-cause mortality (*p* = 0.22) or graft failure (*p* = 0.13).

When Kaplan Meier curves were generated comparing anti-apoA-1 IgG seropositive versus anti-apoA-1 IgG seronegative RTR (Supplementary Figure S1), the same associations were retrieved, namely significance for CVD mortality (*p* = 0.035), but not for all-cause mortality or for incident graft failure.

At the pre-specified cut-off for anti-apoA-1 IgG positivity, sensitivity was 18.0% (95% CI: 9–32), specificity 89.3% (95% CI: 86–92), positive predictive value 17.0% (95% CI: 9–30) and negative predictive value 90.0% (95% CI: 87–92) for CVD-related deaths.

Finally, as shown in Table 3 Cox regression analyses showed that anti-apoA-1 IgG levels were significantly associated with CVD mortality in a model adjusted for age and gender (model 1, HR: 1.56, *p* = 0.002). This association remained significant, independent of adjustment either for the Framingham risk score (FRS, model 2, HR: 1.56, *p* = 0.002), eGFR (model 3, HR: 1.54, *p* = 0.004) or both parameters combined (model 4, HR: 1.54, *p* = 0.004), HDL CEC (model 5, HR: 1.54, *p* = 0.003), history of MI (model 6, HR: 1.45, *p* = 0.0013), primary renal disease (model 7, HR: 1.53, *p* = 0.005) and time between transplantation and baseline (model 8, HR: 1.56, *p* =0.002). Importantly, FRS itself was not associated with CVD mortality in our RTR cohort (unadjusted HR: 1.00 [0.99–1.02], *p* = 0.51; age and gender adjusted HR: 1.00 [0.98–1.03], *p* =0.95). For all-cause mortality, the same associations were retrieved (models 3 and 4; Table 3). On the other hand, no significant associations were detected between anti-apoA-1 IgG levels and incident graft failure (Table 3). In our sensitivity analyses there was no association between anti-apoA-1 IgG levels and non-CVD mortality, further supporting the specificity of the relationship between anti-apoA-1 IgG and CVD mortality (Table 3).

When Cox regression analyses were performed according to anti-apoA-1 IgG seropositivity , the aforementioned associations remained unchanged, at the exception of all-cause mortality which remained significant after adjusting for renal function, and for which the association became close to significance after adjusting for previous MI on top of age and gender (Supplementary Table S2). Again, no association between anti-apoA-1 IgG levels and graft failure could be observed (Supplementary Table S2).

## 4. Discussion

The novel finding of this prospective study is that anti-apoA-1 IgGs are an independent predictor of CVD mortality in a RTR cohort with a follow-up of 7 years. Our observations indicate that traditional CVD risk factors were presently not associated with CVD mortality. Furthermore, no association was observed between anti-apoA-1 IgG and non-CVD mortality in the preformed sensitivity analysis. This reinforces both the possible clinical relevance and the CVD specificity of the present findings. Indeed, to the best of our knowledge and at the exception of renal function markers [3,4,5,21,22], no specific biomarkers of CVD outcome independent of renal function have been identified so far in RTR. Considering the absence of currently validated tools for CVD risk prediction in RTR, a rule-out test with a 90% NPV could conceivably be of clinical interest as a first step in the field of CV risk stratification in these patients. In this context, we hypothesize that a simple standard follow-up could be particularly well adapted to RTR patients with low anti-apoA-1 IgG values. Further validation studies are now required to challenge this hypothesis before any clinical recommendations can be made.

The second notable finding of this study is that anti-apoA-1 IgG were not associated with graft failure, nor with HDL CEC. Although further reinforcing the specificity between anti-apoA-1 IgG and CV outcomes, these results were somehow unexpected, as anti-apoA-1 IgG have been previously shown to be associated with impaired HDL CEC [9,10], lately reported as being an independent predictor of incident graft failure [21]. The reasons for such differences are still elusive, most likely numerous, and possibly related to pathophysiological differences between CVD and graft atherosclerosis. Indeed, rupture of vulnerable atherosclerotic plaques is known to underlie most cases of acute CVD events, while this is not thought to play an important role in chronic transplant vasculopathy-induced graft failure, where progressive arteriolar luminal narrowing due to the intimal accumulation of degenerating smooth muscle-like cells and adventitial fibrosis represent the major pathogenic processes [23]. Furthermore, another explanation could lie in the fact that RTR represent a unique patient population in terms of oxidative stress exposure and persistent loss of HDL function, when compared to e.g. systemic lupus erythematosus patients [10] or dyslipidaemic subjects with preserved renal function [9]. Finally due to the heterogeneity of methodological protocols underlying the numerous unstandardized HDL functional assays, we cannot exclude that an analytical difference between our HDL assay and those from other groups could undermine the present observation [24,25,26]. Therefore, further studies are warranted to determine if this absence of correlation between anti-apoA-1 IgG and HDL functionality in RTR is intrinsically disease-specific.

Thirdly, this study strengthens previous observations and provides the first insights of the anti-apoA-1 IG architecture in RTR. Indeed, the association between these antibodies with previous MI has been consistently reported across different populations with preserved renal function [7,8,11,13]. Reproducing this association reinforces the notion that a previous acute coronary event is an important acquired factor, that could, together with niacin therapy [9] and genetic determinants [27], contribute to better understand the reasons underlying the existence of anti-apoA-1 IgG in individuals without overt signs of clinical autoimmunity. In this context, we report for the first time specific associations with primary glomerular disease and tubulo-interstitial disease as primary renal diseases possibly associated to the existence of anti-apoA-1 IgG.

Lastly, the somewhat lower than expected prevalence of anti-apoA-1 IgG seropositivity retrieved presently (11.5%) when compared to maintenance hemodialysis patients and the general population (20%) [8,18], is worth a comment, as we would have expected an increased prevalence as previously reported in all other clinical situations with a high CV risk [7,8,10,12,13,14,18]. A conceivable explanation for this observation might be that RTRs are under chronic immunosuppressive medication, known to improve features of autoimmunity and thus decrease autoantibody levels. The trend toward a decrease in the prevalence of proliferation inhibitors along the increasing anti-apoA-1 IgG tertiles may lend weight to this hypothesis and warrants further investigations.

Although the results of the present study lend further weight to the growing body of evidence indicating that humoral autoimmunity contributes to CVD, we could not explore the mechanisms by which anti-apoA-1 IgG levels may associate with CVD in RTR. So far, previous animal and in-vitro studies showed that anti-apoA-1 IgG could be active mediators of atherogenesis, inducing myocardial necrosis and death in mice through toll-like receptors (TLR) [2,4] and CD14 heterodimer signaling [6,7,11,15,16,17,28]. Since these deleterious effects could potentially be amended by immunomodulation therapies, either using a specific apoA-1 mimetic peptide or intravenous immunoglobulins, anti-apoA-1 IgGs have been proposed as emergent therapeutic targets [11,29]. In accordance with these in vitro and animal experiments, a functional CD14 polymorphism was recently shown to be a strong modulator of anti-apoA-1 IgG-related CVD risk prediction in the general population [12]. As CD14 expressing monocytes display higher TLR2 and 4 expression in RTR [30], knowing whether CD14 and/or TLR2/4 polymorphisms together with the presence of anti-apoA-1 IgG could further improve prognosis assessment in RTR remains to be investigated. Given the important pathophysiological differences between atherogenesis and transplant vasculopathy [23], knowing whether the aforementioned molecular mechanisms could also explain the increased CV risk ascribed to these antibodies in RTR remains unknown and constitutes an important limitation of the present study. Further, despite the relatively large number of included RTR in this adequately powered study, the number of events was still somewhat low, leading to restricted possibilities with regards to statistical analysis. Since this investigation was also carried out in a single center, further validation of our findings in a larger multicentre cohort appears desirable. In addition, it would be interesting to analyse whether RTR with high anti-apoA-1 IgG titers show a differential response to an intervention with cardiovascular treatment strategies. A further limitation resides in the fact that we did not measure other autoantibodies of possible CV relevance, such as auto-antibodies to β2 glycoprotein I domain I and IV, cardiolipin, heat-shock protein 60, and to phosphorylcholine. Because anti-apoA-1 IgG were shown to display the strongest and independent prognostic accuracy for major adverse cardiovascular events in non-autoimmune settings when compared to the aforementioned auto-antibodies [31], we focused our work specifically on this class of antibodies. Knowing whether the present association could be reproduced with other auto-antibodies remains to be shown. Also, before utilizing anti-apoA-1 IgGs as clinical biomarker, it would be interesting to screen kidney graft donors to learn, whether intra-individual variability in titers has a potential impact on outcomes after transplantation.

In conclusion, we report anti-apoA-1 IgG as a novel prognostic biomarker for CVD mortality in RTR, independent of traditional CVD risk factors and HDL functionality. These data indicate that anti-apoA-1 IgG holds potential as a clinical biomarker for CVD risk stratification in RTR patients, a high CVD risk population with altered functionality of the immune system. Further investigations to define the potential usefulness of anti-apoA-1 IgG assessments in clinical decision making are required, as well as studies to delineate the intrinsic pathophysiological pathways that these antibodies activate to subsequently result in an increased CVD risk in RTR. 

## Figures and Tables

**Table 1:** Baseline characteristics according to gender stratified tertiles of anti-apoA-1 IgG.

Characteristic	Gender Stratified Tertiles of Anti-apoA-1 Levels			*p*-Value
	First (*n* = 154)	Second (*n* = 154)	Third (*n* = 154)	
Recipient demographics				
Anti-apoA-1 IgG, AU (OD_405_ nm)	0.15 (0.11–0.19)	0.31 (0.26–0.36)	0.64 (0.52–0.82)	<0.001
Recipient demographics				
Age, years	50.5 (41.6–59.4)	53.4 (44.7–61.1)	52.1 (44.0–60.8)	0.12
Male gender, *n* (%)	84 (55)	84 (55)	84 (55)	1.00
Current smoking, *n* (%)	28 (18)	33 (21)	22 (14)	0.26
Previous smoking, *n* (%)	70 (46)	67 (44)	72 (47)	0.85
Metabolic syndrome, *n* (%)	83 (57)	94 (65)	84 (60)	0.34
Body composition				
BMI, kg/m^2^	26.1 ± 4.3	26.1 ± 4.2	25.9 ± 4.2	0.86
Lipid Profile				
Total cholesterol, mmol/L	5.6 ± 1.0	5.7 ± 0.9	5.7 ± 1.3	0.49
LDL cholesterol, mmol/L	3.5 ± 1.0	3.6 ± 0.8	3.6 ± 1.2	0.82
HDL cholesterol, mmol/L	1.1 ± 0.3	1.3 ± 0.3	1.1 ± 0.3	0.55
Apolipoprotein A-I, g/L	1.6 ± 0.3	1.6 ± 0.3	1.6 ± 0.3	0.75
Triglycerides, mmol/L	2.1 (1.3–2.7)	2.1 (1.4–2.5)	2.2 (1.4–2.7)	0.22
Cholesterol efflux percentage	7.3 (6.2–8.4)	7.5 (6.3–8.3)	7.6 (6.5–8.9)	0.11
Use of statins, *n* (%)	79 (51)	88 (57)	74 (48)	0.27
Cardiovascular disease history				
History of MI, *n* (%)	12 (7)	8 (5)	20 (13)	0.047
TIA/CVA, *n* (%)	5 (3)	8 (5)	9 (6)	0.54
Blood pressure				
Systolic blood pressure, mmHg	152.2 ± 23.9	151.0 ± 21.4	154.1 ± 22.0	0.47
Diastolic blood pressure, mmHg	89.8 (± 9.8)	89.0 (± 9.5)	90.1 (± 10.0)	0.59
Use of ACE inhibitors, *n* (%)	55 (36)	49 (32)	58 (38)	0.55
Use of *β*–blockers, *n* (%)	90 (58)	93 (60)	95 (61)	0.84
Use of diuretics, *n* (%)	59 (38)	75 (49)	63 (41)	0.16
Number of antihypertensive drugs, *n (*%*)*	2 (1–3)	2 (1–3)	2 (1–3)	0.16
Glucose homeostasis				
Glucose, mmol/L	4.9 (4.1–5.0)	4.8 (4.1–5.0)	4.8 (4.1–5.1)	0.69
Insulin, μmol/L	11.3 (8.7–16.5)	10.6 (7.8–14.8)	11.4 (7.6–15.4)	0.16
HbA1c, %	6.3 (5.8–6.9)	6.3 (5.8–7.0)	6.4 (5.7–7.1)	0.47
HOMA-IR	3.1 (1.7–3.6)	2.7 (1.5–3.4)	2.8 (1.5–3.4)	0.21
Post-Tx diabetes mellitus, *n* (%)	24 (15)	29 (19)	29 (19)	0.69
Use of anti-diabetic drugs, *n* (%)	17 (11)	25 (16)	21 (14)	0.41
Use of insulin, *n* (%)	7 (5)	9 (6)	13 (8)	0.36
Inflammation				
hsCRP, mg/L	3.4 (0.7–4.4)	3.3 (0.9–4.1)	3.3 (1.0–4.2)	0.43
Framingham risk score	17.2 (7.6–27.3)	20.8 (9.6–32.9)	20.7 (8.6–31.3)	0.28
Donor demographics				
Age, years	37.6 (23.0–50.0)	37.2 (23.8–50.0)	37.1 (23.0–51.3)	0.76
Male gender, *n* (%)	76 (49)	90 (59)	85 (56)	0.24
Living kidney donor, *n* (%)	28 (18)	17 (11)	15 (10)	0.06
(Pre)transplant history				
Dialysis time, months	34.8 (12.0–48.3)	37.0 (14.8–51.0)	33.6 (12.8–45.0)	0.44
Primary renal disease				
Primary glomerular disease, *n* (%)	35 (23)	40 (26)	52 (34)	0.08
Glomerulonephritis, *n* (%)	11 (7)	6 (4)	12 (8)	0.32
Tubulo-interstitial disease, *n* (%)	33 (21)	24 (16)	17 (11)	0.05
Polycystic renal disease, *n* (%)	26 (17)	31 (20)	24 (16)	0.56
Dysplasia and hypoplasia, *n* (%)	7 (5)	8 (5)	2 (1)	0.15
Renovascular disease, *n* (%)	11 (7)	12 (8)	6 (4)	0.32
Diabetic nephropathy, *n* (%)	3 (2)	2 (1)	9 (6)	0.04
Other or unknown cause, *n* (%)	28 (18)	31 (20)	32 (21)	0.84
Immunosuppressive medication				
Daily prednisolone dose, mg	9.2 (7.5–10.0)	9.1 (7.5–10.0)	9.1 (7.5–10.0)	0.33
Calcineurin inhibitors, *n* (%)	120 (78)	126 (82)	124 (81)	0.68
Proliferation inhibitors, *n* (%)	124 (81)	109 (71)	108 (70)	0.07
Renal allograft function				
Creatinine clearance, mL/min	47.3 ± 14.6	48.2 ± 15.9	46.5 ± 16.1	0.62
Urinary protein excretion, g/24 h	0.3 (0.0–0.3)	0.2 (0.1–0.2)	0.4 (0.1–0.4)	0.07

Normally distributed continuous variables are presented as mean ± SD. Continuous variables with a skewed distribution are presented as median [25th to 75th percentile]. Categorical data are summarized by *n* (%), and differences were tested by chi-squared test. MI, myocardial infarction; TIA, transient ischemic attack; CVA, cerebrovascular accident; ACE, angiotensin-converting enzyme; Tx, transplantation.

**Table 2:** Multivariate linear regression for baseline characteristics that are significantly associated with anti-apoA-1 IgG in a univariate linear regression.

Characteristics	Unstandardized Coefficient	95% CI	Standardized Coefficient	*p*-Value
Primary glomerular disease	0.086	0.016–0.156	0.116	0.016
History of MI	0.121	0.015–0.227	0.103	0.026
Tubulo-interstitial disease	−0.0.96	−0.182–−0.010	−0.106	0.028

Variables are listed in decreasing order of strength of association. *R*^2^ = 0.043 (Cox & Snell). Model *x*^2^: 49.8; *p* < 0.001.

**Table 3:** Hazard ratios for cardiovascular mortality, all-cause mortality, and graft failure per one standard deviation increase of anti-apoA-1 IgG.

	CVD Mortality		All–Cause Mortality		Graft Failure		Non-CVD Mortality Sensitivity Analysis	
	HR [95%CI] per 1–SD Increase	*p*	HR [95%CI] per 1–SD Increase	*p*	HR [95%CI] per 1–SD Increase	*p*	HR [95%CI] per 1–SD Increase	*p*
**Model 1**	1.56 [1.17–2.07]	0.002	1.36 [1.09–1.70]	0.007	1.17 [0.93–1.48]	0.18	1.41 [0.95–2.09]	0.09
**Model 2**	1.56 [1.17–2.08]	0.002	1.36 [1.09–1.70]	0.007	1.18 [0.94–1.49]	0.16	1.41 [0.94–2.11]	0.09
**Model 3**	1.54 [1.15–2.06]	0.004	1.32 [1.05–1.67]	0.017	1.14 [0.91–1.42]	0.26	1.39 [0.92–2.08]	0.11
**Model 4**	1.54 [1.15–2.07]	0.004	1.32 [1.04–1.66]	0.020	1.15 [0.92–1.44]	0.22	1.39 [0.92–2.10]	0.12
**Model 5**	1.54 [1.16–2.05]	0.003	1.36 [1.09–1.71]	0.007	1.19 [0.94–1–50]	0.15	1.44 [0.98–2.11]	0.06
**Model 6**	1.45 [1.08–1.94]	0.013	1.32 [1.05–1.66]	0.016	1.14 [0.90–1.45]	0.27	1.39 [0.90–2.14]	0.14
**Model 7**	1.53 [1.14–2.05]	0.005	1.33 [1.06–1.67]	0.016	1.17 [0.93–1.48]	0.18	1.45 [0.96–2.20]	0.08
**Model 8**	1.56 [1.18–2.09]	0.002	1.37 [1.09–1.17]	0.07	1.17 [0.93–1.48]	0.18	1.47 [0.98–2.23]	0.07

Model 1: adjustment for recipient age and gender; model 2: model 1 + adjustment for FRS; model 3: model 1 + adjustment for eGFR; model 4: model 1 + adjustment for FRS and eGFR; model 5: model 1 + adjustment for cholesterol efflux capacity; model 6: model 1 + adjustment for history of MI; model 7: model 1 + adjustment for primary renal disease; model 8: model 1 + adjustment for time between transplantation and baseline. In sensitivity analysis non-CVD deaths were used as endpoint. One standard deviation is equivalent to 0.316. HR: Hazard ratios; FRS: Framingham risk score.

## Supplementary Materials

Supplementary materials can be found at https://www.mdpi.com/2077-0383/8/7/948/s1. Table S1: Baseline characteristics according to seropositivity of anti-apoA-1 IgG, Table S2: Hazard ratios for cardiovascular disease mortality, all-cause mortality, and graft failure by seropositivity of anti-apoA1 IgG, Figure S1: Seropositivity of anti-apoA-1 IgG is associated with increased cardiovascular mortality in renal transplant recipients.
