# Supplementary material for: Autoantibodies to Apolipoprotein A-1 as Independent Predictors of Cardiovascular Mortality in Renal Transplant Recipients
Source: J Clin Med. 2019 Jun 29;8(7):948. doi: 10.3390/jcm8070948 (PMC6679113; doi:10.3390/jcm8070948)
Supplement: Supplementary file 1 [file jcm-08-00948-s001.pdf]

Method

Determination of Anti-apoA-1 IgG

Maxisorp plates (NuncTM, Roskilde, Denmark) were coated with purified, human delipidated apoA-1 (20 µg/mL; 50 µL/well) for 1 h at 37 °C. After being washed, all wells were blocked for 1 h with 2% bovine serum albumin (BSA) in a phosphate buffer solution (PBS) at 37 °C. Patient samples were additionally also added to a non-coated well, in order to assess individual non-specific binding. After six washing cycles, a 50 µL/well of signal antibody (alkaline phosphatase-conjugated anti-human IgG; Sigma-Aldrich, St Louis, MO, USA), diluted 1:1000 in a PBS/BSA 2% solution, was added and incubated for 1 h at 37 °C. After washing six more times, phosphatase substrate p-nitrophanylphosphate disodium (Sigma-Aldrich) dissolved in a diethanolamine buffer (pH 9.8) was added and incubated for 20 min at 37 °C (Versa Max, Molecular Devices™, San Jose, CA, USA). Finally, optical density (OD) was determined at 405 nm in duplicate. Corresponding non-specific binding was subtracted from the mean OD for each sample. Specificity of our ELISA to detect antibodies against native and lipid low human apoA-1 was previously confirmed by western blot and tandem-mass spectrometry analyses [1]. The validated cut-off for anti-apoA-1 IgG positivity was prospectively set at an OD cut-off of OD > 0.64, and a percentage of the positive control value above 37% [1–5].

The current anti-apoA-1 assay has been extensively validated with respect to pre- analytical and analytical factors[6], including its specificity to detect polyclonal autoantibodies against lipid-free and unmodified human apoA1 as demonstrated by an orthogonal LC-MS/MS approach coupled with peptide engineering. These studies demonstrated that human anti-apoA-1 IgG polyclonal response is biased against the C-terminal part of the native protein without any post-translational modification [1,7,8].

Determination of Cholesterol Efflux Capacity

In order to determine cholesterol efflux capacity (CEC), THP-1 human monocytes were differentiated into macrophages as previously described[9]. After loading with acetylated LDL and ^{3}H-cholesterol, 2% apoB-depleted plasma was added to induce efflux[9]. Medium was collected after 6 hours and centrifuged to pellet cellular debris. An aliquot of medium was counted to quantitate the effluxed cholesterol label. The radioactivity remaining within the cells was also determined by liquid scintillation counting. Efflux per well is expressed as the percentage of counts released into the medium related to the total dose of radioactivity initially present. Values obtained from control cells without added apoB-depleted patient plasma were subtracted to correct for unspecific efflux.

Statistical Analysis

Renal transplant recipients were divided into gender stratified tertiles based on levels of anti-apoA-1 IgG, and differences of baseline characteristics were tested between these groups. Categorical values are given as absolute numbers (percentages) and differences were tested by chi-squared test. Normally distributed continuous variables are given as mean ± standard deviation and differences were tested by one-way analysis of variance (ANOVA), followed by Bonferroni post hoc test. Skewed continuous variables are presented as median [25th to 75th percentile] and differences between groups were tested by Kruskal-Wallis test, followed by Mann-Whitney U-test.

Thereafter, multivariable linear regression analysis was performed to evaluate which variables predict the levels of anti-apoA-1. Baseline characteristics with a *p*-value of ≤0.2 between tertiles of anti-apoA-1 IgG were first fitted into a univariate analysis in order to avoid over fitting. Variables that had a significant association with anti-apoA-1 IgG in a univariate model were then entered into a multivariate linear regression.

The association of anti-apoA-1 IgG levels with the primary endpoints was assessed by the log-rank test and by Cox proportional hazards regression. Kaplan-Meier curves analyses were

performed across anti-apoA-1 IgG tertiles and according to anti-apoA-1 IgG seropositivity, based upon a predefined and validated anti-apoA-1 IgG cut-off value (an OD value a >0.64 and a percentage of the positive control above 37%). [7,8,11–14,18] Differences were assessed using a log-rank test. Cox regression analyses were used to calculate hazard ratios (HR), reported with their 95% confidence intervals (95%CI). Univariate and multivariate Cox regression analyses were performed per standard deviation (SD) increase of anti-apoA-1 IgG levels, and according to anti-apoA-1 IgG seropositivity. Multivariate analyses were performed using different models, taking into account traditional CV risk factors, renal function, HDL functionality, and all the variables that had significant association with anti-apoA-1 levels in linear regression. The association of anti-apoA-1 was adjusted for recipient age and gender (model 1), Framingham risk score (FRS) (model 2), eGFR (model 3), for FRS and eGFR combined (model 4), CEC (model 5), history of MI (model 6) and primary renal disease (model 7).

Schoenfeld residuals test was used to test the proportional hazard assumption for the outcomes of CVD mortality, all-cause mortality and graft failure for analysis per standard deviation increase ($p = 0.18$, $p = 0.20$ and $p = 0.69$ respectively) and for analysis with seropositivity ($p = 0.38$, $p = 0.09$ and $p = 0.77$ respectively). Sensitivity (SN), specificity (SP), positive predictive and negative values (PPV and NPV, respectively) for anti-apoA-1 IgG seropositivity were computed. = p-values < 0.05 were considered statistically significant. All statistical analyses were performed using the Statistical Package for the Social Sciences version 24 (IBM SPSS) and GraphPad Prism version 6.0.

References

1. Antiochos, P.; Marques-Vidal, P.; Virzi, J.; Pagano, S.; Satta, N.; Hartley, O.; Montecucco, F.; Mach, F.; Kutalik, Z.; Waeber, G.; et al. Impact of CD14 polymorphisms on anti-apolipoprotein A-1 IGG-related coronary heart disease prediction in the general population. *Atherosclerosis* **2017**, *263*, e45.
2. Vuilleumier, N.; Bas, S.; Pagano, S.; Montecucco, F.; Guerne, P.-A.; Finckh, A.; Lovis, C.; Mach, F.; Hochstrasser, D.; Roux-Lombard, P.; et al. Anti-apolipoprotein A-1 IgG predicts major cardiovascular events in patients with rheumatoid arthritis. *Arthritis. Rheum.* **2010**, *62*, 2640–2650.
3. Vuilleumier, N.; Rossier, M.F.; Pagano, S.; Python, M.; Charbonney, E.; Nkoulou, R.; James, R.; Reber, G.; Mach, F.; Roux-Lombard, P. Anti-apolipoprotein A-1 IgG as an independent cardiovascular prognostic marker affecting basal heart rate in myocardial infarction. *Eur. Heart J.* **2010**, *31*, 815–823.
4. Vuilleumier, N.; Montecucco, F.; Spinella, G.; Pagano, S.; Bertolotto, M.; Pane, B.; Pende, A.; Galan, K.; Roux-Lombard, P.; Combescure, C.; et al. Serum levels of anti-apolipoprotein A-1 auto-antibodies and myeloperoxidase as predictors of major adverse cardiovascular events after carotid endarterectomy. *Thromb. Haemost.* **2013**, *109*, 706–715.
5. Pruijm, M.; Schmidtko, J.; Aho, A.; Pagano, S.; Roux-Lombard, P.; Teta, D.; Burnier, M.; Vuilleumier, N. High prevalence of anti-apolipoprotein/A-1 autoantibodies in maintenance hemodialysis and association with dialysis vintage. *Ther. Apher. Dial.* **2012**, *16*, 588–594.
6. Frias, M.A.; Virzi, J.; Batuca, J.; Pagano, S.; Satta, N.; Delgado Alves, J.; Vuilleumier, N. ELISA methods comparison for the detection of auto-antibodies against apolipoprotein A1. *J. Immunol. Methods* **2019**, *469*, 33–41.
7. Pagano, S.; Gaertner, H.; Cerini, F.; Mannic, T.; Satta, N.; Teixeira, P.C.; Cutler, P.; Mach, F.; Vuilleumier, N.; Hartley, O. The Human Autoantibody Response to Apolipoprotein A-I Is Focused on the C-Terminal Helix: A New Rationale for Diagnosis and Treatment of Cardiovascular Disease? *PLoS ONE* **2015**, *10*, e0132780.
8. Teixeira, P.C.; Ducret, A.; Ferber, P.; Gaertner, H.; Hartley, O.; Pagano, S.; Butterfield, M.; Langen, H.; Vuilleumier, N.; Cutler, P. Definition of human apolipoprotein A-I epitopes recognized by autoantibodies present in patients with cardiovascular diseases. *J. Biol. Chem.* **2014**, *289*, 28249–28259.
9. Annema, W.; Dikkers, A.; Freark de Boer, J.; Dullaart, R.P.F.; Sanders, J.-S.F.; Bakker, S.J.L.; Tietge, U.J.F. HDL Cholesterol Efflux Predicts Graft Failure in Renal Transplant Recipients. *J. Am. Soc. Nephrol.* **2016**, *27*, 595–603.

Table S1. Baseline characteristics according to seropositivity of anti-apoA-1 IgG.

	**Baseline Characteristics (*n* = 462)**	**Anti-apoA-1 IgG Positive Patients (*n* = 53)**	**Anti-apoA-1 IgG Negative Patients (*n* = 409)**	***p* Value**
**Recipient demographics**				
Age, years	53.0 [43.4-60.4]	54.6 [44.6-62.1]	52.9 [43.2-60.1]	0.47
Male gender, n (%)	252 (55)	34 (64)	218 (53)	0.14
Current smoking (%)	83 (18)	5 (9)	78 (19)	0.09
Previous smoking, n (%)	209 (45)	26 (49)	183 (45)	0.53
Metabolic syndrome, n (%)	261 (60)	29 (60)	232 (57)	0.98
**Body composition**				
BMI kg/m^2	26.0±4.2	25.9±4.6	26.0±4.18	0.77
**Lipid Profile**				
Total cholesterol, mmol/L	5.6±1.1	5.6±1.0	5.6±1.1	0.98
LDL cholesterol, mmol/L	3.5±1.0	3.5±0.9	3.6±1.0	0.83
HDL cholesterol, mmol/L	1.1±0.3	1.1±0.3	1.1±0.3	0.75
Apolipoprotein A-I, g/L	1.6±0.3	1.6±0.3	1.6±0.3	0.33
Triglycerides, mmol/L	1.9 [1.4-2.6]	1.9 [1.4-2.7]	1.9 [1.4-2.6]	0.59
Cholesterol efflux (%)	7.5±1.7	7.6 ±1.5	7.5±1.7	0.76
Use of statins, n (%)	241 (52)	24 (45)	241 (52)	0.29
**Cardiovascular disease**				
**history**	40 (9)	9 (17)	31 (8)	0.02
History of MI, n(%)	22 (5)	1 (2)	21 (5)	0.30
TIA/CVA, n (%)				
**Blood pressure**	152.4±22.5	154.9±20.9	152.1±22.7	0.40
Systolic blood pressure,	89.7±9.9	90.9±8.8	89.5±10.0	0.36
mmHg	162 (35)	23 (43)	139 (34)	0.18
Diastolic blood pressure,	278 (60)	33 (62)	245 (60)	0.74
mmHg	197 (43)	21 (40)	176 (43)	0.64
Use of ACE inhibitors, n (%)	2.0 [1-3]	2.0 [1-3]	2.0 [1-3]	0.53
Use of *β*-blockers, n (%)				
Use of diuretics, n (%)	4.5 [4.1-5.0]	4.4 [4.0-5.3]	4.5 [4.1-5.0]	0.45
Number of antihypertensive	11.1 [7.9-15.2]	11.2 [6.5-17.5]	11.0 [8.0-15.2]	0.74
drugs, n (%)	6.3 [5.8-6.9]	6.4 [5.6-7.0]	6.3 [5.8-6.9]	0.97
**Glucose homeostasis**	2.3 [1.6-3.4]	2.1 [1.4-4.0]	2.3 [1.6-3.4]	0.58
Glucose, mmol/L	82 (18)	12 (23)	70 (17)	0.32
Insulin, μmol/L	63 (14)	9 (17)	54 (13)	0.45
HbA1C, %	29 (6)	5 (9)	24 (6)	0.31
HOMA-IR				
Post-Tx diabetes mellitus, *n*	1.9 [0.8-4.1]	2.12 [0.6-3.9]	1.87 [0.8-4.2]	0.83
(%)	0.3 [0.2-0.5]	0.9 [0.7-1.3]	0.3 [0.2-0.4]	<0.001
Use of anti-diabetic drugs, *n*	19.1 [8.6-30.4]	19.6 [8.0-34.5]	19.0 [8.7-29.9]	0.29
(%)				
Use of insulin, *n (%)*	39.0 [23.0-51.0]	42.0 [28.0-54.0]	37.0 [23.0-50.0]	0.05
**Inflammation**	251 (55)	24 (46)	227 (56)	0.20
hsCRP, mg/L	60 (13)	8 (15)	52 (13)	0.63
Anti-apoA-1 IgG, AU (OD_{405}				
nm)	27.0 [13.0-47.3]	29.0 [12.0-44.5]	27.0 [13.0-48.0]	0.99
**Framingham risk score**				
**Donor demographics**	127 (28)	21 (40)	106 (26)	0.03
Age, year	29 (6)	5 (9)	24 (6)	0.31
Male gender, *n (%)*	74 (16)	3 (6)	71 (17)	0.03
Living kidney donor, *n (%)*	81 (18)	5 (9)	76 (19)	0.10
**(Pre)transplant history**	17 (4)	0 (0)	17 (4)	0.13
Dialysis time, months	29 (6)	4 (8)	25 (6)	0.69
**Primary renal disease**	14 (3)	3 (6)	11 (3)	0.24
Primary glomerular disease,	91 (20)	12 (23)	79 (19)	0.57
n (%)				

Glomerulonephritis, *n (%)*	10 [7.5-10]	10.0 [7.5-10]	10.0 [7.5-10.0]	0.84
Tubulo-interstitial disease, *n*	370 (80)	46 (87)	324 (79)	0.19
(%)	341 (74)	37 (70)	304 (74)	0.48
Polycystic renal disease, *n*				
(%)	47.5±15.7	46.4±14.5	47.5±15.7	0.63
Dysplasia and hypoplasia, *n*	0.1 [0.1-0.3]	0.1 [0.1-0.5]	0.1 [0.0-0.3]	0.04
(%)				
Renovascular disease, *n (%)*				
Diabetic nephropathy, *n (%)*				
Other or unknown cause, *n (%)*				
**Immunosuppressive medication**				
Daily predisolone dose, mg				
Calcineurin inhibitors, *n (%)*				
Proliferation inhibitors, *n (%)*				
**Renal allograft function**				
eGFR, mL/min				
Urinary protein excretion, g/24 h				

Normally distributed continuous variables are presented as mean ± SD, and differences were tested with students t-test. Continuous variables with a skewed distribution are presented as median [25th to 75th percentile], and differences were tested by Mann–Whitney U test. Categorical data are summarized by *n* (%), and differences were tested by chi-squared test. TIA, transient ischemic attack; CVA, cerebrovascular event; ACE, angiotensin-converting enzyme; Tx, transplantation.

Table S2. Hazard ratios for cardiovascular disease (CVD) mortality, all-cause mortality, and graft failure by seropositivity of anti-apoA1 IgG.

	**CVD Mortality**		**All-cause Mortality**		**Graft Failure**	
	**HR [95%CI]**	***p* Value**	**HR [95%CI]**	***p* Value**	**HR [95%CI]**	***p* Value**
Model 1	3.32 [1.47–7.52]	0.004	2.26 [1.17–4.39]	0.016	1.50 [0.70–3.20]	0.30
Model 2	3.33 [1.47–7.55]	0.004	2.26 [1.16–4.39]	0.016	1.46 [0.68–3.12]	0.34
Model 3	3.30 [1.45–7.49]	0.004	2.24 [1.15–4.37]	0.018	1.48 [0.69–3.19]	0.31
Model 4	3.29 [1.45–7.47]	0.005	2.22 [1.14–4.33]	0.019	1.48 [0.69–3.19]	0.32
Model 5	3.75 [1.60–8.78]	0.002	2.54 [1.28–5.03]	0.007	1.53 [0.71–3.27]	0.28
Model 6	2.57 [1.09–6.05]	0.031	1.91 [0.96–3.77]	0.065	1.13 [0.44–2.91]	0.80
Model 7	3.08 [1.36–7.02]	0.007	2.11 [1.08–4.10]	0.028	0.88 [0.35–2.23]	0.78
Model 8	3.36 [1.49–7.59]	0.004	2.30 [1.19–4.45]	0.014	1.49 [0.69–3.18]	0.31

Model 1: adjustment for recipient age and gender; model 2: model 1 + adjustment for FRS; model 3: model 1 + adjustment for eGFR; model 4: model 1 + adjustment for FRS and eGFR; model 5: model 1 + adjustment for cholesterol efflux capacity. Function; model 6: model 1 + adjustment for history of MI; model 7: model 1 + adjustment for primary renal disease; model 8: model 1 + adjustment for time between transplantation and baseline.

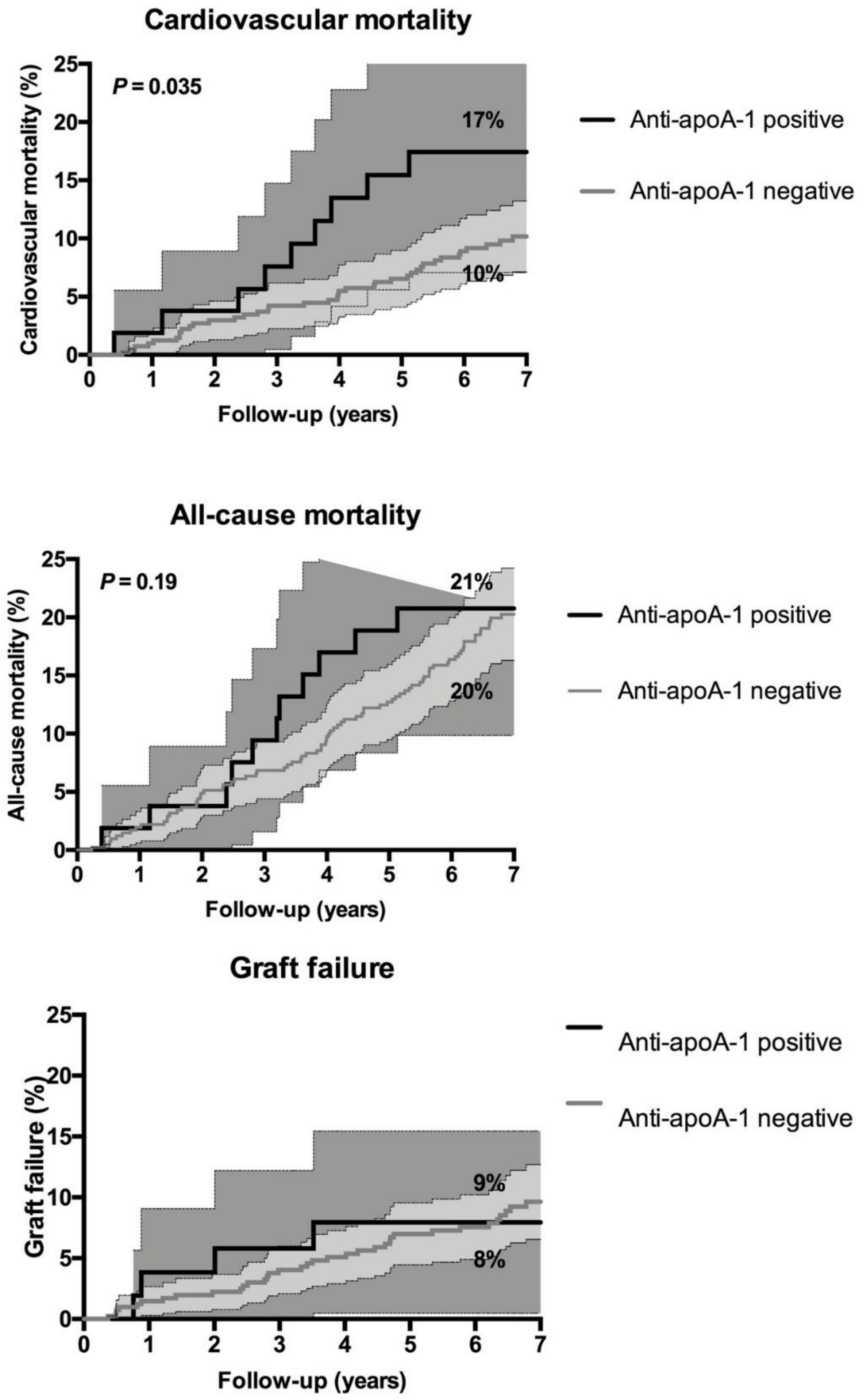

Figure S1. Seropositivity of anti-apoA-1 IgG is associated with increased cardiovascular mortality in renal transplant recipients. Kaplan-Meier curves of (**A**) cardiovascular mortality, (**B**) all-cause mortality, and (**C**) graft failure according to positivity of anti-apoA-1 IgG. The corresponding *p* value was obtained from log-rank tests.
